# Mechanisms of mechanical load transfer through brain tissue

**DOI:** 10.1038/s41598-023-35768-3

**Published:** 2023-05-29

**Authors:** Nina Reiter, Friedrich Paulsen, Silvia Budday

**Affiliations:** 1 Institute of Continuum Mechanics and Biomechanics, Friedrich-Alexander-Universität Erlangen-Nürnberg, Egerlandstr. 5, 91058 Erlangen, Germany; 2 Institute for Functional and Clinical Anatomy, Friedrich-Alexander-Universität Erlangen Nürnberg, Universitätsstr. 19, 91054 Erlangen, Germany

**Keywords:** Biomedical engineering, Biophysics, Soft materials, Mechanical engineering

## Abstract

Brain injuries are often characterized by diffusely distributed axonal and vascular damage invisible to medical imaging techniques. The spatial distribution of mechanical stresses and strains plays an important role, but is not sufficient to explain the diffuse distribution of brain lesions. It remains unclear how forces are transferred from the organ to the cell scale and why some cells are damaged while neighboring cells remain unaffected. To address this knowledge gap, we subjected histologically stained fresh human and porcine brain tissue specimens to compressive loading and simultaneously tracked cell and blood vessel displacements. Our experiments reveal different mechanisms of load transfer from the organ or tissue scale to single cells, axons, and blood vessels. Our results show that cell displacement fields are inhomogeneous at the interface between gray and white matter and in the vicinity of blood vessels—locally inducing significant deformations of individual cells. These insights have important implications to better understand injury mechanisms and highlight the importance of blood vessels for the local deformation of the brain’s cellular structure during loading.

## Introduction

Brain tissue is highly sensitive to mechanical impacts. Mechanically induced brain injuries are usually characterized by diffusely distributed axonal and vascular damage. While axonal damage is often invisible to the imaging techniques used in medical diagnosis^1^, vascular damage can show as micro bleeds^2,3^ or lead to blood-brain barrier breakdown^4,5^. Computational simulations of brain injury are a valuable tool to provide more insights into the spatial distribution of harmful stresses and strains during head impacts. Previous studies have shown that the distribution of high stresses and strains obtained from computational simulations correlates well with the damage distribution seen in histologically analyzed injured brains^6–9^.

However, while computational models can help to identify brain regions that experience particularly high mechanical loadings, they fail to explain why within those vulnerable regions some cells are damaged while neighboring cells remain unaffected. It is still unclear how the forces acting on the brain at the tissue scale are transferred to cells, cellular processes such as axons and dendrites, and blood vessels^10^.

Previous studies have provided first insights into the effects of mechanical loading on the brain’s microstructure^11^. Existing investigations assessing damage mechanisms associated with traumatic brain injury (TBI) have mostly used animal models, for example performing controlled cortical impact (reviewed in^12^), or performed sequential mechanical testing and histological examination on animal tissue^13,14^, in vitro cell cultures^15,16^, or brain organoids^17^. Bar-Kochba *et al.*^18^ simultaneously loaded and imaged neurons embedded in a 3D collagen gel. Still, to the best of the authors’ knowledge, there have not been experiments with simultaneous tissue-scale mechanical loading and microstructural observations of native brain tissue, although the latter are key to understand the dynamic processes and mechanisms of load transfer taking place during deformation.

**Figure 1:**
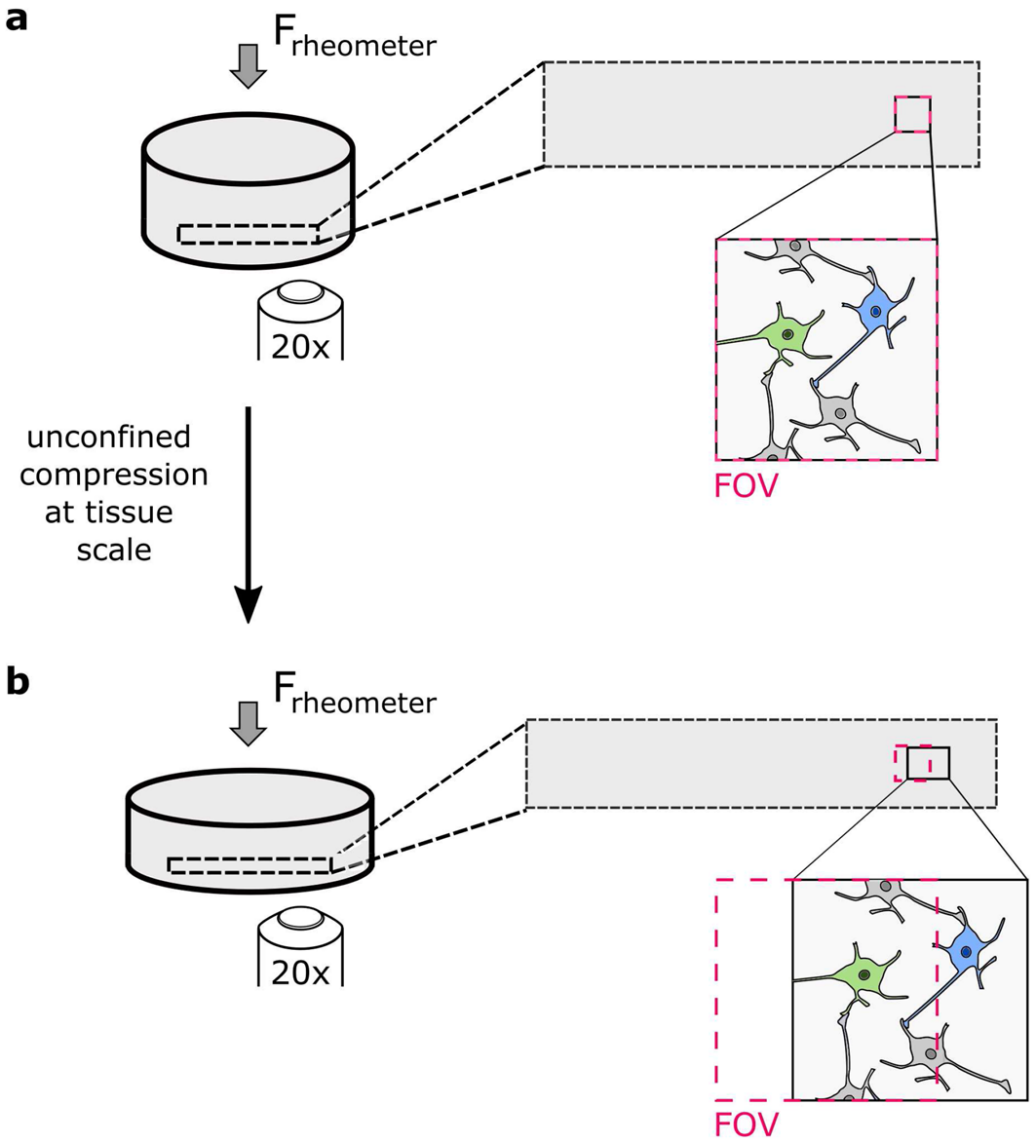
Initial (**a**) and deformed (**b**) configuration of a cylindrical tissue sample. When the sample is compressed, it widens, which leads to tensile deformations in the plane perpendicular to the force F (elongation of the rectangle indicated with black dotted lines). The microscope objective is placed under the specimen, its field of view (FOV) is indicated with red dotted lines. The blue neuron leaves the field of view when the specimen is deformed. The elements in the figure are not true to scale.

Here, we subjected histologically stained fresh human and porcine brain tissue specimens to compressive loading at the tissue scale and simultaneously observed the resulting displacements of cells and blood vessels through an inverse microscope, as schematically shown in Fig. 1, to reveal mechanisms of mechanical load transfer through brain tissue.

## Results

### Influence of histological staining on tissue mechanics

Supplementary Fig. S1 shows the average response of porcine brain tissue samples during cyclic compression loading before and after histological staining. We stained and tested $n=4$ specimens for each dye and the control group, respectively. Our results show that specimens stained with resorcinol fuchsine are significantly stiffer after staining than during the initial experiment. Those samples also had a stiff and brittle surface after staining that easily ruptured during handling. The specimens stained with methylene blue or nuclear fast red are marginally softer after staining, similar to the control specimens.

Since methylene blue had the least effect on the mechanical tissue response of all tested dyes and because it not only marks cell nuclei but also neuronal cell bodies—making it possible to distinguish neurons from glial cells—, we chose this dye for all subsequent experiments.

### Homogeneous cell displacement fields

**Figure 2:**
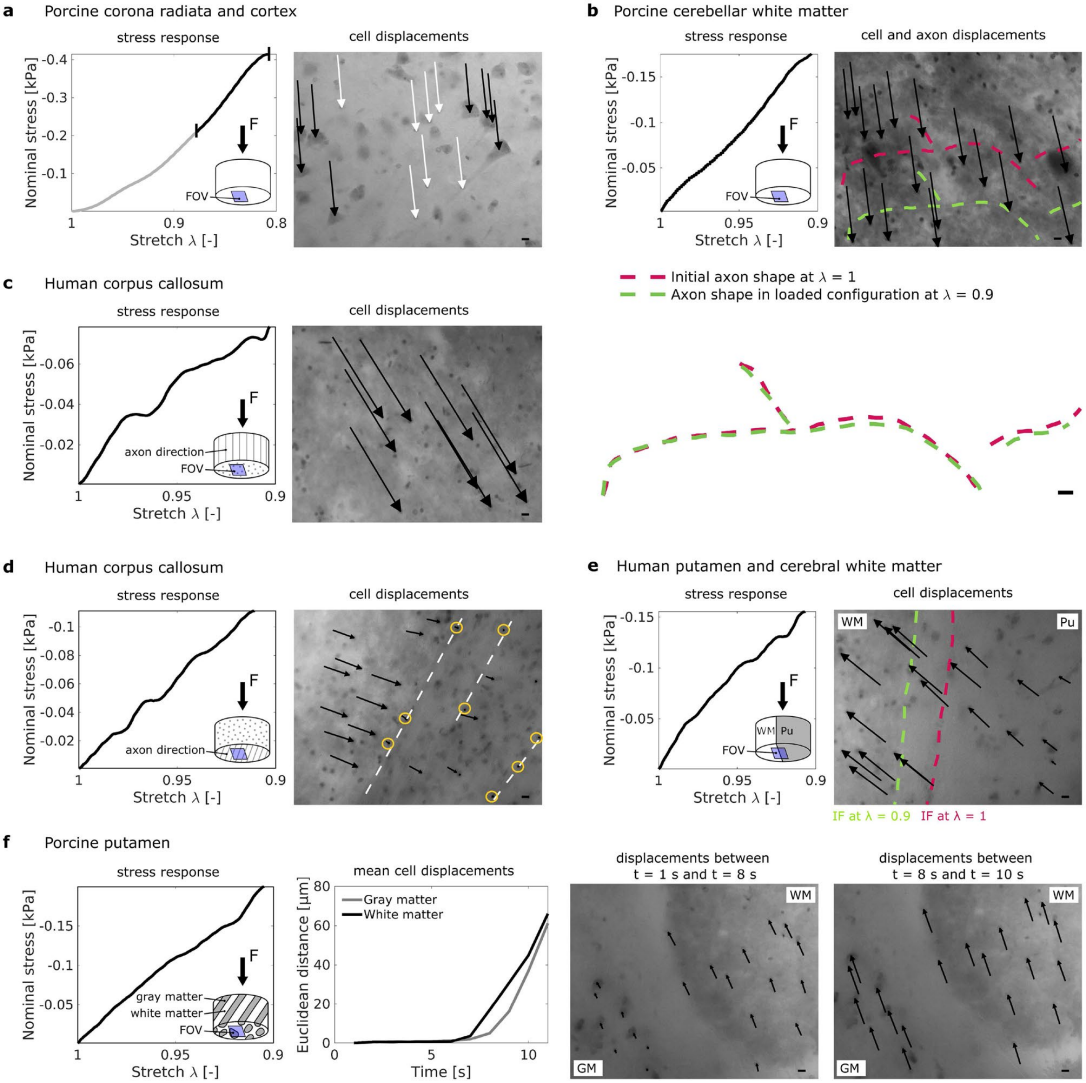
Homogeneous (**a**–**c**) and inhomogeneous (**d**–**f**) cell displacement fields in human and porcine brain samples. (**a**) Homogeneous displacements of neurons (right image, black arrows) and glial cells (right image, white arrows) were tracked only for the second part of the loading phase (black interval in the stress response shown on the left). (**b**–**f**) Arrows in the microscope images point from the initial location of selected cell nuclei to their final location at maximum strain (deformation) $\varepsilon _{max} = 1-\lambda _{min} = 10$%. Sub-figure (**b**) shows displacements of cells and axons (red dotted line $=$ initial axon location, green dotted line $=$ final axon location). The overlay of the axon shapes before and after loading displayed below shows no significant differences in orientation and shape. (**c**–**d**) Cell displacements in the corpus callosum depend on whether the sample is loaded along (**c**) or transverse (**d**) to its main axon direction. White dotted lines in the microscope image in (**d**) indicate large axons, yellow circles emphasize cells that remained almost at their initial location. (**e**) Close to the interface (IF) between white matter (WM) and putamen (Pu) in a human brain sample, cell displacements within the putamen were inhomogeneous. (**f**) In the porcine putamen, cells within the white matter started moving earlier and initially faster than cells within the gray matter. For samples with inhomogeneous tissue, the location of the field of view (FOV) with respect to the inhomogeneities is indicated schematically (not true to scale). Scale bars $= 10$ µm.

In most of the samples, the cell displacements were homogeneous within the field of view, i.e., all cells moved in the same direction and covered the same distance, so that inter cellular distances remained constant, as exemplarily shown in Fig. 2a. Neurons and glial cells were displaced in the same way during loading, i.e., there were no relative motions between the network of neurons and the network of glial cells.

In one sample from the cerebellum, large myelinated axons were visible in the recorded videos. The axons were lighter than the surrounding tissue, presumably because their lipid containing myelin sheaths repelled the water-based methylene blue. In Fig. 2b, those axons are marked with dotted lines. An overlay of the traced fibers in the unloaded and loaded state shows that the axons are displaced during loading without changing their shape and orientation, in accordance with the homogeneous displacement field of surrounding cells.

### Inhomogeneous cell displacement fields in the human corpus callosum

Figure 2c,d shows how glial cells in samples from the human corpus callosum are displaced during uniaxial compression. The sample in Fig. 2c was loaded along the main axon direction, while the sample in Fig. 2d was loaded transverse to the main axon direction. In the sample loaded along its main axon direction, the cell displacement field is homogeneous and cells are displaced over a mean distance of $125.4 \pm 4.6$ µm. In the sample loaded transverse to its main axon direction, cells are displaced heterogeneously. Cells located close to large axons almost remain at their initial location; cells distributed between the large axons are displaced perpendicular to the main axon direction and cover distances ranging from 8 to 42 µm. The measured stress in this sample was slightly higher than in the sample loaded along its main fiber direction.

### Inhomogeneous cell displacement fields at gray-white matter interfaces

Figure 2e shows how cells are displaced at the interface between putamen and white matter tissue in a human brain tissue sample. During loading, the interface shifted towards the left side of the image. The cells within white matter tissue were displaced almost homogeneously and covered a mean distance of $59.6 \pm 3.6$ µm. Simultaneously, the cells within the putamen moved heterogeneously and covered distances between 6 and 50 µm. The locally inhomogeneous tissue deformation leads to changes in the intercellular distance of up to $50\%$.

Figure 2f shows the stress response and cell displacements in a sample from the porcine putamen. In contrast to the human putamen with a homogeneous appearance at the tissue scale, the porcine putamen consists of thin layers of gray and white matter and thus has many internal gray-white matter interfaces. One of those interfaces was within the field of view of the microscope, as indicated in the first sub-figure of Fig. 2f. During loading, cells in white matter started moving earlier and initially faster than cells in gray matter tissue.

### Cell body deformation during initial loading

**Figure 3:**
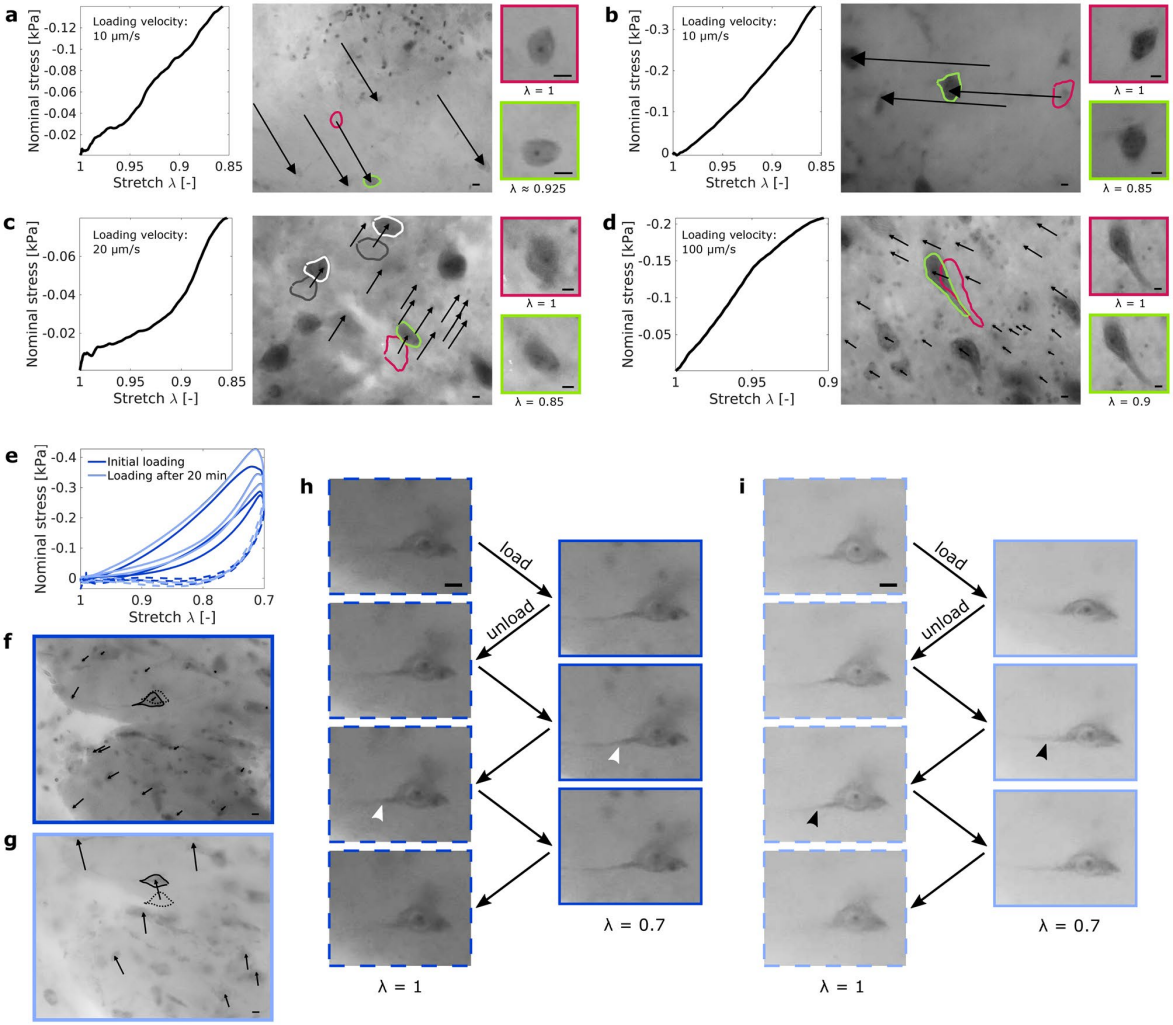
Cell body deformation during initial (**a**–**d**) and repetitive (**e**–**i**) loading. (**a**) shows a deforming cell body in a sample from the porcine putamen and (**b**–**d**) show deforming cell bodies in the porcine brain stem. Arrows point from the initial location of selected cell nuclei to their final location at maximum strain (deformation) $\varepsilon = 1-\lambda$. Red lines indicate the initial shape of a deforming neuron, green lines the deformed shape at maximum strain. The quadratic images show magnified views of the undeformed (red frame) and deformed (green frame) neurons. In (**c**), two neurons that remained undeformed are exemplarily shown (gray line $=$ initial shape, white line $=$ shape at maximum strain). (**e**–**i**) show the deformation of a cell body in the porcine putamen during repetitive cyclic compression. The sample was able to recover its initial stiffness after a 20 min recovery period (**e**), but the displacement field after recovery (**g**) was different from the displacement field during the initial experiment (**f**). In (**f**) and (**g**), black arrows point from the initial location of cell nuclei to their final location at maximum strain (deformation) $\varepsilon _{max} = 1-\lambda _{min} = 30$%. The neuron shown in (**h**) remained undeformed during the initial loading. One of its neurites is visible (white arrowheads) and does not change its course during loading. After the recovery period, the shape of the neuron and the angle of its neurite (black arrowheads) changed under loading (**i**). The dark blue color indicates the initial experiment, the lighter shade of blue the second experiment after recovery. Dashed lines represent the unloaded state, solid lines the loaded state. Scale bars $=$ 10 µm.

In 166 videos, only eight cell bodies deformed visibly. In three of the samples with deforming cells, the local displacement field was notably inhomogeneous. Figure 3a–d present the stress-deformation response and the corresponding microstructural analyses for four porcine brain specimens in which a cell body within the field of view was deformed during loading. In all four specimens, the deforming neuron was surrounded by other cells that remained undeformed during the experiment. Two of the undeformed cells are exemplarily shown in Fig. 3c. In the first three cases (a–c), the displacement field was homogeneous around the deforming neuron. However, in Fig. 3a and c, the deforming neuron was displaced less than the surrounding cells. In these two cases, the shape of the deforming neuron changed significantly during loading and our observations indicate a certain rotation. The neuron shown in Fig. 3b clearly changed its orientation during loading as the angle between the soma and the bottom neurite changes.

In Fig. 3d, the displacement field around the large deforming neuron was inhomogeneous. In this case, the cell body is deformed due to the gradient in the local deformation of the surrounding tissue.

### Cell body deformation during repetitive loading

Figure 3e–i shows a neuronal cell body in a specimen from the porcine putamen that was loaded repetitively. The neuron shows no visible deformation during an initial set of three loading cycles, but is deformed when the experiment is repeated after a 20 min recovery period. Figure 3e indicates that the mechanical tissue response is fully restored after the recovery period. However, the local displacement field during the first loading cycle of the first experiment (Fig. 3f) differs notably from the displacement field during the first loading cycle of the second experiment (Fig. 3g): during the first experiment, the cell nuclei cover a mean distance of $9.9 \pm 5.9$ µm and move towards the lower left corner of the image; during the second experiment, they cover a mean distance of $26.1 \pm 8.8$ µm and move towards the upper edge of the field of view. Figure 3h,i show a magnified view of the neuron during the first and second experiment. During the first three loading cycles, the neuron was not visibly deformed. However, during the second experiment, the shape of the neuron as well as the angle of the left neurite changed noticeably during loading.

### Blood vessel deformation

**Figure 4:**
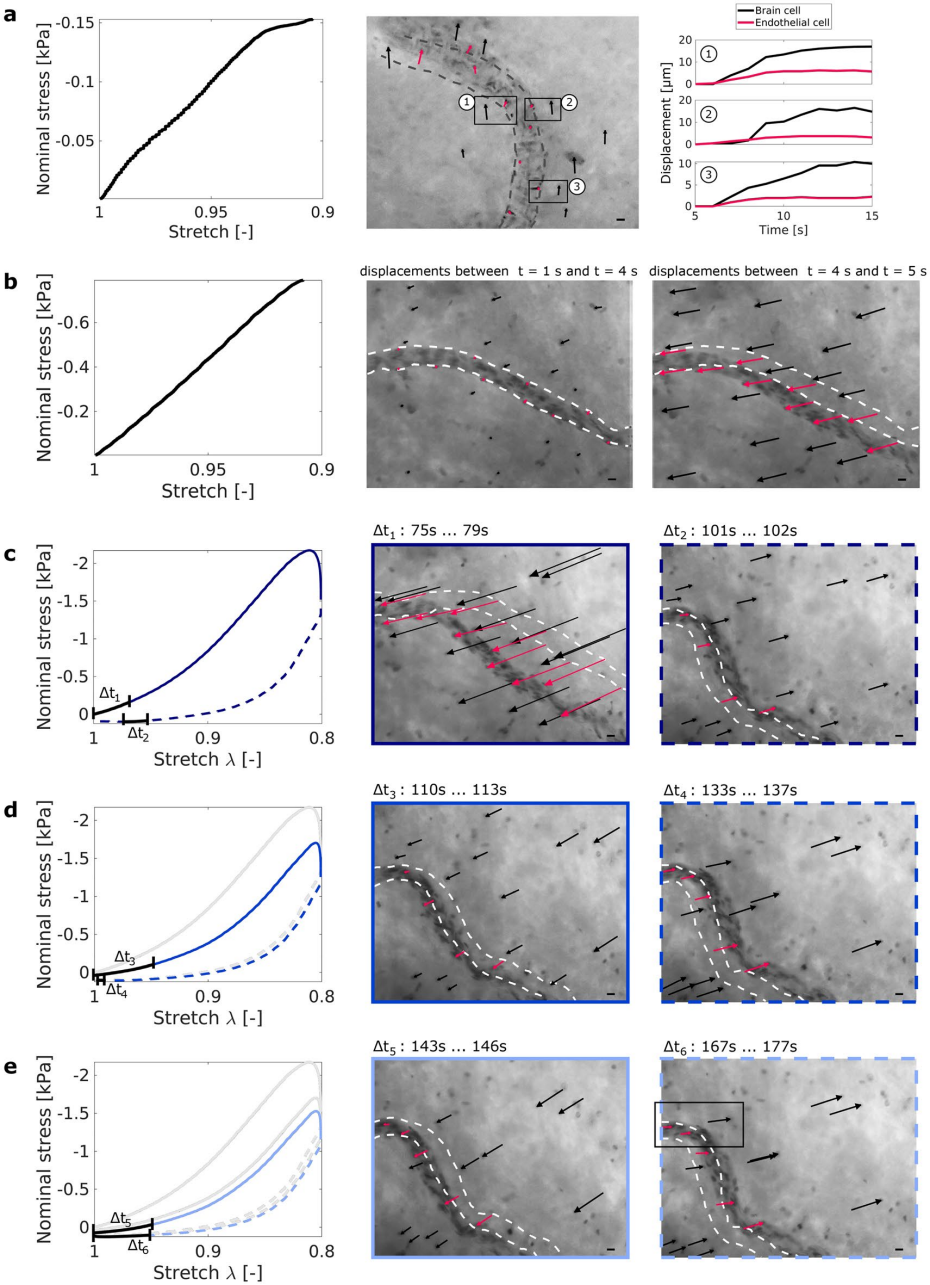
Blood vessel dynamics for small ($\varepsilon _{max} = 10$%) (**a**–**b**) and large ($\varepsilon _{max} = 20$%) (**c**–**f**) strain (deformation) levels, shown for two samples from the porcine corona radiata. In (**a**), brain cells move more than endothelial cells during loading. In the bottom part of the field of view, the blood vessel seems to detach from the surrounding tissue, which leads to a sliding interface. The displacements over time of a brain cell and a neighboring endothelial cell are compared for three pairs of brain and endothelial cells (black boxes). In (**b**), the blood vessel initially resists deformation and brain tissue from the upper part of the image is pushed against the blood vessel. After 4 s, the blood vessel starts moving and cell displacements increase. Black arrows indicate brain cell displacements, red arrows indicate endothelial cell displacements. Gray dotted lines indicate the shape of the blood vessels at the beginning of the experiments. (**c**–**e**) show the same blood vessel as in (**b**) but subjected to a larger strain level with maximum strain $\varepsilon _{max} = 20$%. The blood vessel was displaced out of the field of view (FOV) during loading and could thus not be tracked continuously. The left figure in (**c**) shows the stress response (measured force over sample cross-section area) of the sample depending on the applied sample deformation (stretch). The center and right figures in (**c**) show the deformation of the blood vessel and the cell displacements during the first loading-unloading cycle (black arrows $=$ brain cell displacements, red arrows $=$ endothelial cell displacements). (**d**) and (**e**) show the second and third loading and unloading cycle, respectively. Cell displacements were tracked during the time intervals $\Delta t$ that are indicated as black segments in the stress-stretch curves. Scale bars $=$ 10 µm.

Figure 4a shows a blood vessel during uniaxial compression (10% maximum strain, corresponding loading curve shown on the left) of a sample from the porcine corona radiata. Brain cell displacements (black arrows in the microscope image) around the blood vessel are inhomogeneous. In the bottom part of the field of view, brain cells cover much larger distances than neighboring endothelial cells (red arrows in the microscope image). Figure 4a, right, shows the displacements of three brain cell and endothelial cell pairs over time. The dynamic processes captured in the respective video (Supplementary Video S1) indicate a sliding interface between the vessel wall and the brain parenchyma.

Figure 4b shows a blood vessel inside a porcine brain tissue sample from the corona radiata that was subjected to three cycles of 10% compression (only the first loading cycle is shown in the figure). During loading, the cells in the upper right corner of the field of view start moving earlier than the blood vessel and the cells in the lower left corner (Supplementary Video S2). The cells seem to be pushed against the blood vessel, which is blocking further movement. After four seconds, the blood vessel and the cells in the lower left corner start moving as well.

**Figure 5:**
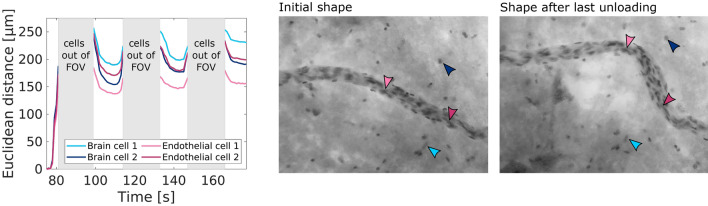
Permanent deformation in a blood vessel subjected to repetitive loading. The blood vessel did not recover its original shape after being subjected to three cycles of 10% and three cycles of 20% compressive strain. The arrowheads on the microscope images indicate two brain cells and two endothelial cells that could still be identified after repeatedly leaving and re-entering the field of view (FOV). The displacements of those cells during the three loading cycles with maximum strain $\varepsilon _{max} = 20$% are shown in the left subfigure.

Figures 4c–e and 5 show the deformation behavior of the same blood vessel as in Fig. 4b, but during compression loading with a maximum of 20% strain (deformation). Figure 4c shows that the blood vessel buckled during the first loading cycle with 20% maximum strain. It did not recover its original shape after unloading. During the second loading cycle (see Fig. 4d), the tissue moved less than before. The cell displacements around the blood vessel were inhomogeneous, i.e., the cells in the upper right corner of the image were displaced more than the cells in the direct vicinity of the blood vessel. The lower part of the blood vessel moved more than the upper part both during loading and unloading. During the third loading cycle (see Fig. 4e), the cell displacements around the blood vessel are again inhomogeneous, similar to the second loading cycle, but even more pronounced—especially during unloading (see varying directions of the black arrows).

The endothelial cells within the black box in Fig. 4e moved less than the neighboring brain cell. This could indicate that the part of the blood vessel that is aligned with the direction of brain cell displacements was detached from the brain parenchyma. Figure 5 further highlights the permanent rearrangements within the tissue after the first loading and differences in the displacements between brain cells and endothelial cells.

## Discussion

**Figure 6:**
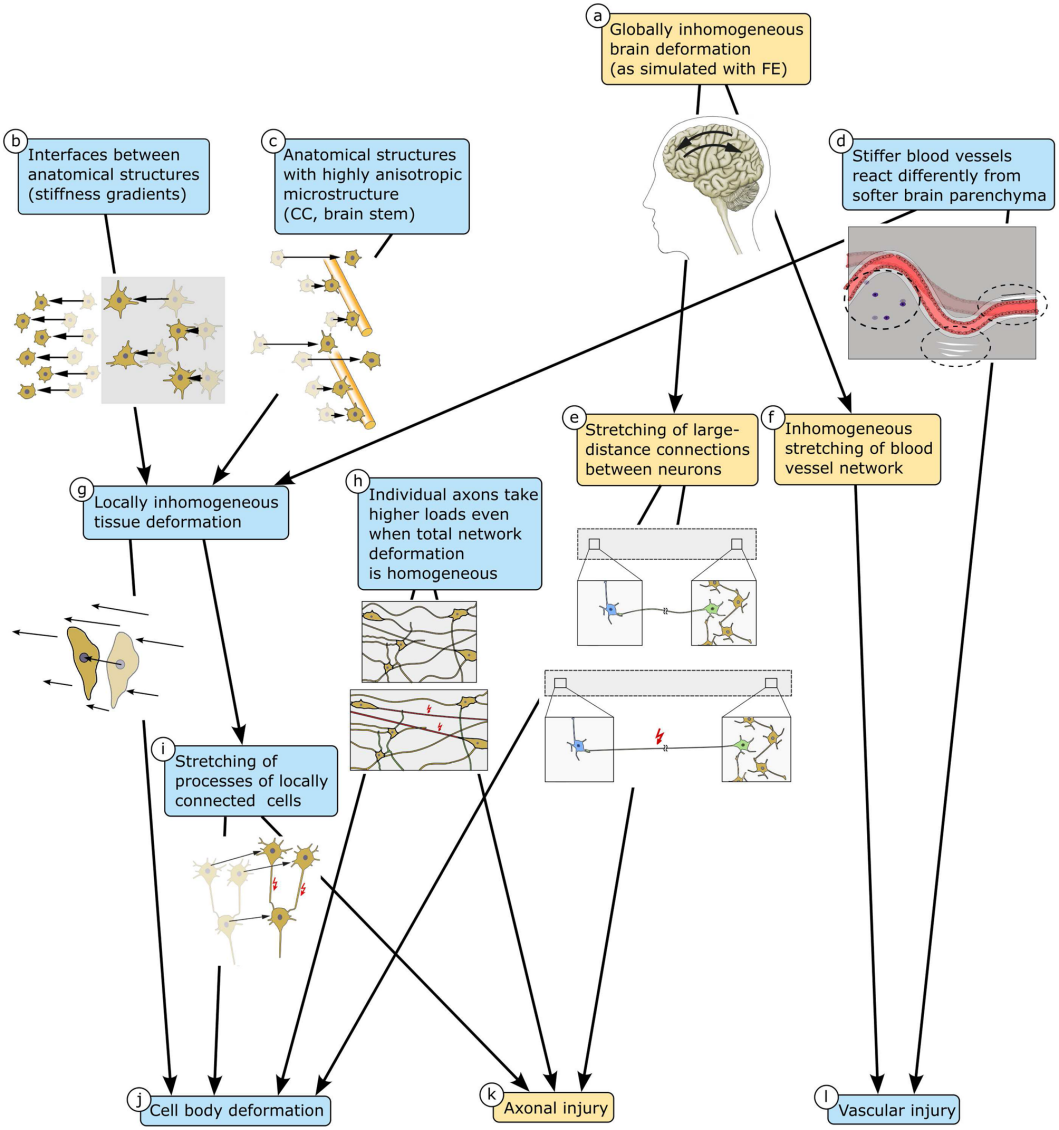
Load transfer mechanisms from the organ to the cell scale. Global brain deformation is usually inhomogeneous (**a**), which leads to stretching of axons that connect neurons over large distances in the brain (**e**) and also causes inhomogeneous stretching of the blood vessel network (**f**). Tensile stretching of axons and blood vessels can lead to axonal (**k**) and vascular (**l**) injury, respectively. Blood vessels are stiffer than the brain parenchyma and therefore react differently to mechanical loading (**d**). The different behavior of blood vessels can lead to locally inhomogeneous tissue deformation (**g**). Locally inhomogeneous tissue deformation also occurs at interfaces between anatomical structures of differing stiffness (**b**) and in fiber tracts such as the corpus callosum (CC) and the brain stem, which have an anisotropic microstructure due to highly aligned axons (**c**). In locally inhomogeneously deforming tissue, large cell bodies may experience deformations that result directly from deformation gradient in the surrounding tissue (**g**–**j**). Additionally, axons of locally connected neurons may be stretched (**i**), which can lead to axonal injury (**k**) or deformation of the affected neurons (**j**). Even when the local tissue deformation is homogeneous, individual axons may take higher loads depending on their orientation (**h**) and thus be more susceptible to injury (**k**). Blue boxes indicate effects we could observe in the presented experiments. Yellow boxes indicate observations from the literature and hypotheses.

To investigate how mechanical loading at the tissue scale is transferred to cells and blood vessels at the cell scale in the human and porcine brain, we have subjected histologically stained fresh tissue samples to compressive loading and simultaneously tracked the resulting displacements of cells and blood vessels through an inverse microscope. Our experiments have revealed the different mechanisms of how organ- or tissue-scale loads are passed down to single cells, axons, and blood vessels, as summarized in Fig. 6.

Our observations show that cells experience large displacements but are hardly deformed upon loading. In homogeneous specimens, all cell nuclei moved uniformly. Only in few specimens, the distance between neighboring cells changed slightly. Interestingly, however, at gray-white matter interfaces, in the vicinity of blood vessels, and in the corpus callosum loaded transverse to its main fiber direction, the cell displacement field was inhomogeneous. This indicates that gradients in the Poisson’s ratios or tissue stiffness (due to interfaces) and embedded stiffer elements (like blood vessels or large axon bundles in the corpus callosum) can lead to inhomogeneous cell displacements, i.e., inhomogeneous deformation states at the cell scale—even for uniaxial loading conditions at the tissue scale. Interestingly, large axons in the porcine cerebellar white matter in Figure 2b do not lead to inhomogeneous displacements. This suggests that the aforementioned effect of stiffer fiber elements is limited to large axon bundles rather than individual axons. Additionally, the regionally varying density of the extracellular matrix and glial cell network can affect the interactions between axons and the surrounding tissue.

Inhomogeneous deformation states at the cell level might lead to local stretching of intercellular connections such as axons, dendrites, and glial processes (see paths b/c/d–g–i in Fig. 6). Our results therefore suggest that regions in the vicinity of blood vessels or gray-white matter interfaces are more vulnerable than others. This is in agreement with recent studies showing amplified stresses in axons near the gray-white tissue interface^19^. Our observations also agree well with a previous study demonstrating that hemorrhagic contusions particularly evolve at the gray-white interface underlying the somatosensory cortex^20^. An additional effect observed through our experiments is that axons in the corpus callosum seem to be damaged more easily when the corpus callosum is stretched perpendicular to the main axon direction. This mechanism could potentially explain why axonal injury is accentuated in caudal parts of the corpus callosum^21^, where axons presumably experience transverse loading during a frontal impact to the forehead.

The observation that cell displacement fields in the corpus callosum were homogeneous when loading occurred along the main axon direction, but inhomogeneous for loading transverse to it could be an indication of transversely isotropic material behavior on the microscale. While several studies have investigated the macroscopic material behavior of the corpus callosum and have found certain differences in the material behavior depending on the loading direction^22–26^, the observed trends mostly showed no clear statistical significance confirming transversely isotropic material behavior on the macroscale, especially for the human brain^25–27^. Therefore, based on our observations, we hypothesize that transversely isotropic behavior at the cellular level of the corpus callosum might influence injury mechanisms, while it is not necessarily relevant for macroscale whole brain simulations.

Only few of the examined cell bodies deformed visibly during compressive mechanical loading. In one case, the deforming cell was large enough to be affected by the gradient in the local deformation (path g–j in Fig. 6). For the other cases, the cell deformation cannot be explained by inhomogeneities in the local tissue deformation alone. But, the neurons might have deformed because their axons or dendrites were stretched. This can happen if (1) the neuron projects over a large distance to a neuron in a region that is displaced in the opposite direction during loading (e.g., if the green neuron in Fig. 1 was connected to a neuron in the left part of the sample) (path a–e–j in Fig. 6), or if (2) the neuron’s axon crosses an inhomogeneously deforming brain region, or if (3) the neuron’s axon or dendrites take loads in the homogeneously deforming tissue because they are aligned with the loading direction (paths h–j and h–k in Fig. 6). We note that as our specimens were quite small compared to the total size of the brain, many large-distance connections were disrupted during specimen preparation. The number of deforming cells might be higher *in vivo* where all connections are intact and able to take loads.

In summary, our observations suggest that cell body deformation under tissue scale mechanical loading is triggered by axon stretching rather than by local deformation of the tissue that surrounds the cell body. Loads applied at the tissue scale seem to be taken by the network of intercellular connections and potentially the extracellular matrix rather than the cell bodies. This observation agrees with^28^ who found that blast TBI leads to decreased dendritic branching and density in the parietal cortex and hippocampus and^29^ who correlated cell density with tensile and compressive stiffness and concluded that cellular connections are more relevant to the mechanical response than the cells themselves. This also indicates that the individual stiffnesses of neuronal and glial cell bodies are not decisive for the tissue stiffness measured at the macroscopic scale.

We also observed significant cell body deformations in a specimen that was subjected to repetitive loading, including two sets of three loading cycles up to 30% compression. Interestingly, the deformations only occurred during the second set of experiments, even though the specimen had fully recovered its initial stiffness (macroscopic mechanical response) at the beginning of the second experiment. This observation indicates that our previously defined limit for brain viscoelasticity of 30% (see^30^), below which no mechanical damage occurred, might only be valid from a macroscopic mechanical point of view: Even if a tissue sample can recuperate its initial macroscopic mechanical response after a recovery period, it may have incurred damage at the microscopic scale. We therefore conclude that the threshold for macroscopic mechanical damage can not necessarily be understood as a threshold for functional brain damage or the integrity of brain cells and their processes.

Finally, our experiments revealed that blood vessel deformations can lead to complex deformation states in their vicinity with inhomogeneous brain cell displacements and considerable relative movements between the blood vessel and the surrounding tissue. At lower strain levels (up to 10%), we attribute inhomogeneous brain cell displacements around blood vessels to the fact that the softer brain parenchyma is pushed against the stiffer blood vessel—which first offers resistance and only starts moving later during loading. The parenchyma on the opposite side of the vessel with respect to the force application is initially ’protected’ from the loading. At higher strain levels (20 % and more), blood vessels can bend and/or buckle and thereby exacerbate local brain tissue deformation. This may lead to changes in the relative positions of cells (axon stretching) or even tissue rupture. The observation that there is a higher risk of tissue rupture and axonal damage in the vicinity of blood vessels agrees well with a study showing that chronic traumatic encephalopathy (CTE) occurs particularly in perivascular regions^31^: Perivascular tau pathology started already at stage I CTE, which indicates that the perivascular space is especially vulnerable, and axons around blood vessels were injured—possibly due to inhomogeneous tissue deformation in these areas. We note that changes in the perivascular space could also result from dysfunction of the blood brain barrier (BBB) due to the disruption of vascular wall integrity caused by the mechanical impact, which leads to influx of inflammatory cells into the traumatized brain parenchyma^32^.

Importantly, blood vessels can detach from the surrounding brain parenchyma (creating a sliding interface), even under strains that are as low as 10%. This indicates that the connecting elements between the vessel wall and the brain parenchyma, i.e., the basement membrane containing extracellular matrix components like fibronectin and laminin, endothelial cells, pericytes, and the endfeet of astrocytes, may be a weak spot of the tissue that can rupture more easily. Our observations are in line with a recent study showing that traumatic microbleeds are not exclusive to moderate or severe TBI but were also identified in mild TBI patients^3^. Interestingly, in our experiments, detachment of the blood vessels occurred where the blood vessel was aligned with the direction of brain cell displacements.

Simulations assessing the influence of the vasculature on brain tissue strains during injury^33^ or predicting vascular stresses in brain tissue^34^ and the location of microbleeds^35^ have so far been based on the assumption that there are no relative displacements between blood vessels and the surrounding tissue. According to our experiments, this assumption does not necessarily hold true for strains exceeding about 10%. To the best of the authors’ knowledge, neither a threshold for cerebral vessel rupture nor for detachment of the vessel from the tissue has been identified to date.

Since we tested dead brain tissue samples, we could only observe the passive response of cells and blood vessels. Impairment of brain function might occur before cells and blood vessels are visibly deformed or the tissue is torn. In experiments performed on living neurons in three dimensional collagen gels by^18^, for instance, first signs of cytoskeletal degradation were seen around six hours post injury. Furthermore, the resolution of the microscope objective and camera did not allow us to observe tissue components that are smaller than cell nuclei. Resolving structures on the molecular level is not only hindered by the resolution of the optical system but also by the setup itself: the larger the chosen magnification, the smaller are the dimensions of the field of view, making it more likely for structures of interest to move out of it during the experiment.

In summary, our study highlights the importance of blood vessel deformations and relative movements between the vessels and surrounding brain parenchyma during mechanical tissue loading. Such effects should be considered in the future when assessing brain tissue damage under mechanical impacts. In the following, we summarize the main insights we could obtain through our experiments:

- Most cells move homogeneously within the network of intercellular connections and extracellular matrix, while their cell bodies are hardly deformed $\Rightarrow$ the individual stiffnesses of neuronal and glial cell bodies are not decisive for the tissue stiffness measured at the macroscopic scale.
- Cell displacement fields are inhomogeneous at the interface between gray and white matter, in the vicinity of blood vessels, and in the corpus callosum loaded transverse to its main fiber direction $\Rightarrow$ higher risk of damage.
- Cell bodies deform when their axons or dendrites are stretched or when they experience a gradient in the displacement field.
- The threshold for macroscopic mechanical damage can not necessarily be understood as a threshold for functional brain damage or the integrity of brain cells and their processes.
- Blood vessels can detach from the brain parenchyma during mechanical loading.

## Methods

### Human and porcine brain tissue

For the simultaneous mechanical and microstructural analyses, we used human and porcine brain tissue. We obtained one human brain (see Supplementary Fig. S2A) from a female body donor at the age of 69 who had given her written consent to donate her body to research. The study was approved by the Ethics Committee of Friedrich-Alexander-Universität Erlangen-Nürnberg, Germany, with the approval number 405_18 B. We conducted all procedures in accordance with the Declaration of Helsinki. The body donor had suffered from breast cancer with metastases. During brain dissection, we detected one metastasis in the left cerebellar peduncle. The remaining tissue did not exhibit any visible abnormalities. We extracted two samples from the corpus callosum, where nerve fiber bundles are aligned uniaxially, and one sample from the interface between the putamen and the cerebral white matter, as shown in Figure S2D. We first cut the whole brain into coronal slices of approximately 5 to 15 mm thickness. We then immersed the coronal slices in Ringer’s solution and used a biopsy punch with a diameter of 8 mm to extract cylindrical specimens. In case the resulting samples had a height of more than 6 mm, we carefully shortened them with a surgical scalpel. The specimen heights *H* ranged from 3.3 to 5.3 mm. After extraction, we stained the fresh specimens to visualize microstructural elements, as described in more detail in the next section.

In addition to the human brain, we obtained porcine brain hemispheres from a local slaughterhouse. Before testing, the brain hemispheres were kept refrigerated in an air-tight container to avoid dehydration. We extracted samples from the corona radiata, the putamen, the brain stem, and the cerebellar white matter, as shown in Figure S2. We first cut the cerebral hemisphere into coronal slices of approximately 5 mm thickness. Since the porcine brain samples deformed more significantly under their own weight than the human brain samples, we now first stained the coronal slices with methylene blue and afterwards punched out cylindrical specimens of 8 mm diameter. The heights *H* of the porcine brain samples ranged from 3.8 to 5.3 mm.

All specimens, both from the human and the porcine brains, were prepared right before testing to minimize dehydration and deformation due to gravitational effects.

### Staining of microstructural tissue components

We used histological staining to visualize cells inside the specimens because histological dyes are less sensitive to longer periods of light exposure than fluorescent cell markers. In addition, in contrast to antibody staining that only marks specific structures of interest, histological dyes not only accumulate in the structures of interest but also slightly stain the surrounding tissue, which is an advantage when aiming to understand the tissue’s deformation mechanisms.

While histological staining techniques were developed and optimized for chemically fixed or deep-frozen thin tissue sections, our experiments required fresh tissue samples with unaltered mechanical properties. Therefore, we had to adjust the staining techniques to account for the different absorption behavior of fresh brain tissue. With the typical soak times for fixed tissues, the fresh samples were overstained. With a reduced soak time of 2 min, we obtained a clear stain with nuclear fast red (Carl Roth GmbH, Karlsruhe, Germany), methylene blue (Morphisto GmbH, Frankfurt am Main, Germany), and resorcinol fuchsine (Carl Roth GmbH, Karlsruhe, Germany). Nuclear fast red stains cell nuclei in a bright pink, methylene blue stains nuclei in a dark blue and somata in lighter shades of blue, and resorcinol fuchsine stains elastic fibers, which are mainly found in blood vessels, in a dark purple. All three dyes also slightly stained the tissue between these structures of interest. An exemplary stained human brain sample is shown in Figure S2B.

### Mechanical testing with simultaneous microstructural observation

For mechanical testing, we used a Discovery HR-3 rheometer from TA Instruments (New Castle, Delaware, USA), as illustrated in Figure S2C.

In a first step, we investigated the influence of the three histological stainings established in the previous section on the mechanical tissue response. To this end, we first subjected fresh unstained tissue samples to three cycles of compressive loading with a maximum strain of 10% corresponding to a maximum displacement in *z*-direction $\Delta z = 0.1H$ and a stretch $\lambda =[H+\Delta z]/H=0.9$ (see Fig. S2f). Subsequently, we let the samples recover surrounded by a small amount of phosphate-buffered saline solution (PBS) for 10 min. We then immersed the specimens in the respective dyes (nuclear fast red, methylene blue, resorcinol fuchsine) for 2 min and cleared the staining in PBS. Control specimens were immersed in PBS for 2 min and cleared as well. After clearing, we repeated the initial experiment.

In a second step, we performed mechanical measurements with simultaneous microstructural observation by using the Discovery HR-3 rheometer with the corresponding inverse microscope module, as schematically shown in Fig. 1. As shown in Figure S2C and F in greater detail, the stained cylindrical specimens are placed on a fixed glass plate above a microscope objective. On the top surface, the samples are held by the upper specimen holder of the rheometer. We added PBS to hydrate the samples during testing and to minimize the friction between the sample and the glass plate as well as between the sample and the upper sample holder so that the contact state at the lower and upper surface of the sample can be assumed to be frictionless. During the experiments, the specimen holder is lowered along the *z* axis to compress the sample. As the compressed specimen deforms, cells in its bottom plane are displaced in the *x*–*y* plane. We observed those cell displacements through the microscope objective and recorded videos with a monochromatic camera. We chose a magnification of 20$\times$ since, on the one hand, it allowed us to distinguish individual larger cell bodies and track their deformations, and, on the other hand, it was still small enough so that the observed cells did usually not leave the field of view during the experiments. The resulting field of view has a size of 326.67 $\times$ 245 $\upmu$m. Simultaneously, we record time, force, and displacement data with a data logger provided by TA Instruments.

For the experiments presented in the current work, we used three types of testing protocols in compression: single loading and unloading, three cycles of loading and unloading, and six cycles of loading and unloading with a recovery period between the third and fourth cycle. The applied stretches range from 0.9 to 0.7.

### Data analysis

Time, gap and force data measured by the rheometer were analyzed using custom-written MATLAB codes. Image data were analyzed as follows: firstly, we extracted relevant frames from the recorded videos; then, we tracked cell coordinates manually; the resulting coordinates and time data were finally analyzed with a custom-written MATLAB code.

### Ethical and consent

This study was approved by the Ethics Committee of Friedrich-Alexander-Universität Erlangen-Nürnberg, Germany, with the approval number 405_18 B. The body donor had given her written consent to donate her body to research. We conducted all procedures in accordance with the Declaration of Helsinki.

## Supplementary Information

Supplementary Information 1.

Supplementary Video 1.

Supplementary Video 2.

## Data Availability

The datasets generated and/or analyzed during the current study are available from the corresponding author on reasonable request.
