# Supplementary material for: Mechanisms of mechanical load transfer through brain tissue
Source: Sci Rep. 2023 May 29;13:8703. doi: 10.1038/s41598-023-35768-3 (PMC10227083; doi:10.1038/s41598-023-35768-3)
Supplement: Supplementary file 1 — Supplementary Information 1. [file 41598_2023_35768_MOESM1_ESM.pdf]

Supplementary Information: Mechanisms of mechanical load transfer through brain tissue

Nina Reiter[a], **Friedrich Paulsen**[b], **and Silvia Budday**[a,✉]

[a]Department of Mechanical Engineering, Friedrich-Alexander-Universität Erlangen Nürnberg, Egerlandstr. 5, 91052 Erlangen, Germany
[b]Institute for Functional and Clinical Anatomy, Friedrich-Alexander-Universität Erlangen Nürnberg, Universitätsstr. 19, 91054 Erlangen, Germany

Supplementary figures

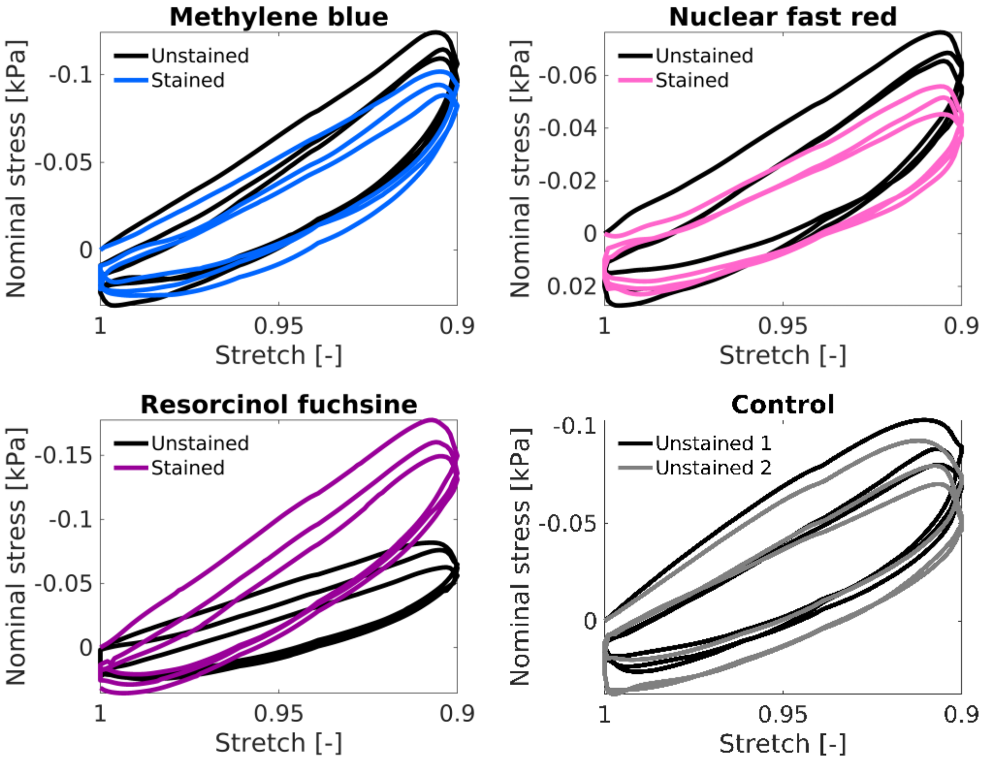

Fig. S1. Influence of three histological stains on the mechanical response of brain tissue. First, the unstained specimens were subjected to three compression cycles (black curves). Subsequently, we let the samples recover for 10 min before staining and testing them again (colored curves). The control samples were subjected to the same procedure but soaked in PBS instead of dye.

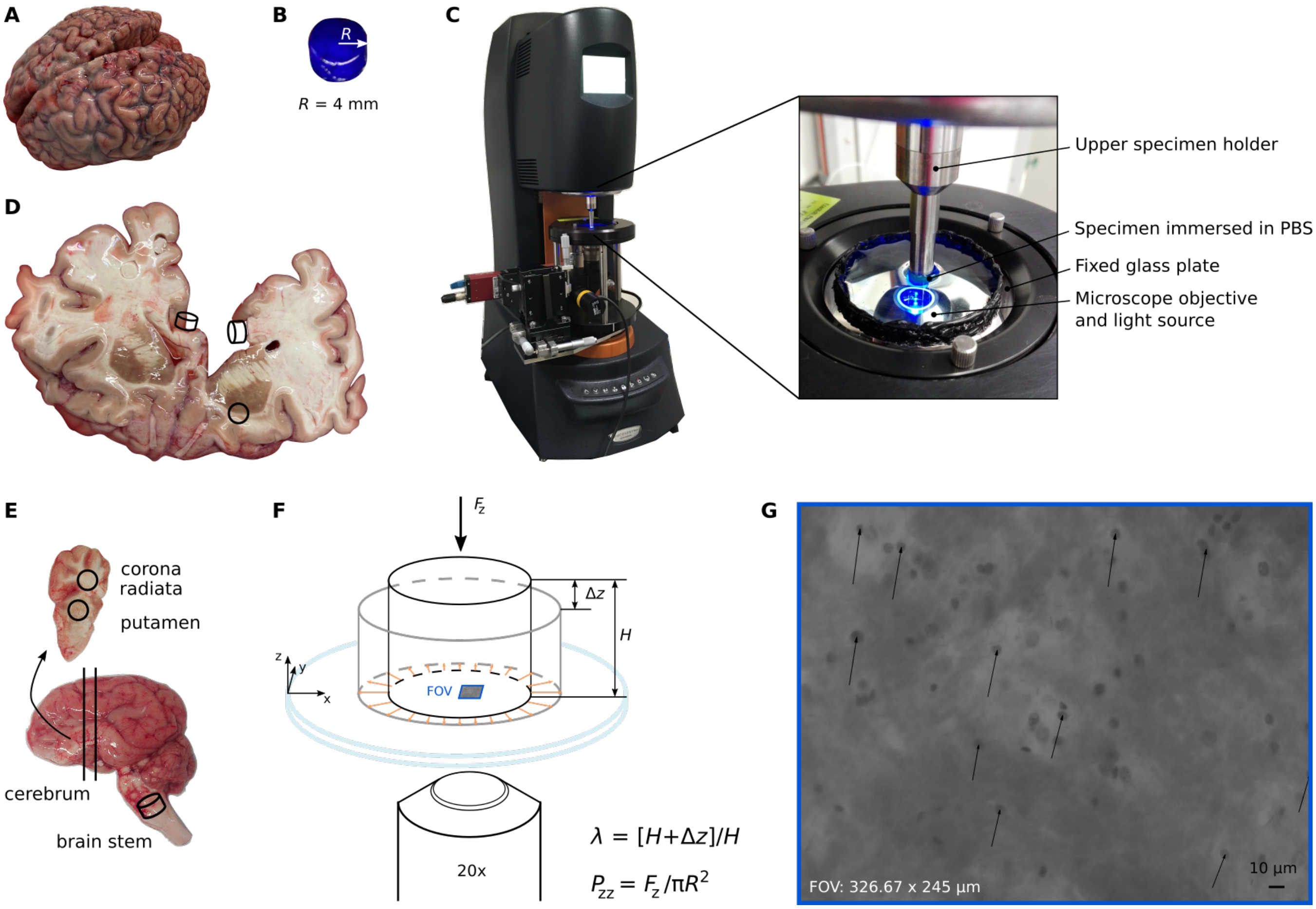

Fig. S2. Specimen preparation and experimental setup. Before staining, cylindrical specimens (B) are extracted from human (A) and porcine (E) brains at different locations as shown in (D) and (E). The specimens with the height H are mounted to a rheometer with microscope module (C) and loaded through the force F_z in z direction, which results in nominal stresses P_{zz} , as shown in (F). The field of view (FOV, shown in G) for cell tracking lies in the $x-y$ plane.

Supplementary movies

Movie S1. Deforming blood vessel during loading and unloading of a tissue sample from the porcine corona radiata. The upper part of the blood vessel is displaced while the lower part does not move. The brain tissue surrounding the lower part of the blood vessel is detached and displaced in y direction, creating a sliding interface between the blood vessel and the brain tissue.

Movie S2. Deformation of a blood vessel during loading of a tissue sample from the porcine corona radiata. Brain tissue is initially pushed against the non-moving blood vessel. Shortly after, the blood vessel eases and is displaced with the surrounding brain tissue.
